# The Influence of Lanthanum Admixture on Microstructure and Electrophysical Properties of Lead-Free Barium Iron Niobate Ceramics

**DOI:** 10.3390/ma17153666

**Published:** 2024-07-25

**Authors:** Dariusz Bochenek, Dagmara Brzezińska, Przemysław Niemiec, Lucjan Kozielski

**Affiliations:** Institute of Materials Engineering, Faculty of Science and Technology, University of Silesia in Katowice, 75 Pułku Piechoty 1a, 41-500 Chorzów, Poland; przemyslaw.niemiec@us.edu.pl (P.N.); lucjan.kozielski@us.edu.pl (L.K.)

**Keywords:** lead-free materials, perovskites, permittivity, dielectric loss

## Abstract

This article presents the research results of lead-free Ba_1−3/2*x*_La*_x_*(Fe_0.5_Nb_0.5_)O_3_ (BFN*x*La) ceramic materials doped with La (*x* = 0.00–0.06) obtained via the solid-state reaction method. The tests of the BFN*x*La ceramic samples included structural (X-ray), morphological (SEM, EDS, EPMA), DC electrical conductivity, and dielectric measurements. For all BFN*x*La ceramic samples, the X-ray tests revealed a perovskite-type cubic structure with the space group P*m*$\bar{3}$*m*. In the case of the samples with the highest amount of lanthanum, i.e., for *x* = 0.04 (BFN4La) and *x* = 0.06 (BFN6La), the X-ray analysis also showed a small amount of pyrochlore LaNbO_4_ secondary phase. In the microstructure of BFN*x*La ceramic samples, the average grain size decreases with increasing La content, affecting their dielectric properties. The BFN ceramics show relaxation properties, diffusion phase transition, and very high permittivity at room temperature (56,750 for 1 kHz). The admixture of lanthanum diminishes the permittivity values but effectively reduces the dielectric loss and electrical conductivity of the BFN*x*La ceramic samples. All BFN*x*La samples show a Debye-like relaxation behavior at lower frequencies; the frequency dispersion of the dielectric constant becomes weaker with increasing admixtures of lanthanum. Research has shown that using an appropriate amount of lanthanum introduced to BFN can obtain high permittivity values while decreasing dielectric loss and electrical conductivity, which predisposes them to energy storage applications.

## 1. Introduction

Perovskite ceramic materials of the general formula ABO_3_ are widely used in modern microelectronic applications, e.g., piezoelectric transducers, microwave frequency resonators, multilayer capacitors, sonar, sensors, and MEMS devices [1,2,3,4,5]. The compounds, most often with the addition of appropriate admixtures obtained based on the Pb(Zr_1−*x*_Ti*_x_*)O_3_ (PZT), have the broadest range of applications due to a high dielectric constant and spontaneous polarization, low dielectric loss, excellent piezoelectric and pyroelectric properties, and a high degree of energy conversion [6,7]. PZT-based ceramic materials are used in a variety of detectors and actuators [5], elements for electric energy harvesting and sensing purposes [8], and ultrasonic rotary inchworm motors [5]. However, environmental concerns due to the toxicity of lead have forced researchers to find alternative materials. Additional enlargement application possibilities are provided by material designs with electromagnetic properties or by combining ferroelectric and magnetic materials (so-called multiferroics) in one material or in a composite form [9,10]. The final properties of multiferroic materials depend mainly on the synergy of their subsystems (e.g., magnetic, electric, and elastic), i.e., on the degree of coupling of individual subsystems [11].

Lead-free materials that could replace PZT compounds are also being sought, but so far it has not been possible to obtain a lead-free ceramic material with such versatile and functional properties as well-known PZT. A group of lead-free ferroelectromagnetic materials and compounds includes BiFeO_3_, Bi_0.5_Na_0.5_TiO_3_, Bi_5_Ti_3_FeO_15_, K_0.5_Na_0.5_NbO_3_, Na_0.5_Bi_0.5_TiO_3_, BaFe_0.5_Nb_0.5_O_3_, Ba_1−*x*_Sr*_x_*TiO_3_, Na_0.5_Bi_0.5_TiO_3_, and K_0.5_Bi_0.5_TiO_3_ [12,13,14,15,16] as well as multi-component ceramic compounds obtained on their basis [17,18,19,20]. Various synthesis methods are used to obtain lead-free ceramic materials, e.g., sol-gel [21,22], combustion synthesis (SHS) [23], the co-precipitation method [24], mechanochemical activation [25], and sintering methods like pressure-less sintering [26,27], hot isostatic pressing (HIP) [28,29], hot pressing (HP) [30], spark plasma sintering (SPS) [31], the cold sintering process (CSP) [32], and the microwave technique [33]. However, not all known synthesis and sintering methods are equally effective in obtaining high values of performance parameters and are suitable for obtaining all lead-free ceramic materials. Therefore, the appropriate selection of technological conditions and the type of synthesis and sintering method used are extremely important to obtain the optimal properties of ceramic materials.

One of the widely studied lead-free ceramic materials is BaFe_0.5_Nb_0.5_O_3_ (BFN). The barium iron niobate material has a perovskite structure with the general formula ABO_3_, where the Ba is placed in the A positions of the structure. At the same time, the Fe/Nb in are placed in the B positions (in an alternating manner). At room temperature RT, the BFN material has a perovskite monoclinic [34] or cubic structure [35]. The non-toxic BFN material belongs to the family of multiferroic materials and shows good dielectric properties (high values of permittivity) [36] and magnetic properties. BFN exhibits relaxor behavior with diffuse phase transition (DPT) and a wide area of the phase transition temperature [18,37,38]. The main negative features of BFN materials include high values of dielectric loss [39], strongly blurred phase transition, and high sintering temperatures (often in the range of 1350–1400 °C) [18]. Different modifiers introduced into the basic compound [40,41,42] as well as various modern synthesis [43,44] and sintering [45,46,47] methods are used in the technological process [43,46,47,48] to reduce the electrical conductivity and to improve the dielectric properties of BFN materials. Auromun et al. [40] doped BFN with a small amount of scandium (Sc), which allowed them to obtain BFN-type ceramics showing good ferroelectric properties. In [49], Intatha et al. presented the results of testing the BFN material doped with lithium fluoride (using low-temperature sintering), obtaining high values of the dielectric constant but with high dielectric loss and no clear maximum of the ferroelectric–paraelectric phase transition. In turn, in [50], BFN was doped with bismuth to lower the sintering temperature, reducing it to 1200–1300 °C, whereas in [41], by doping BFN with Ga, high dielectric constant values were obtained but with equally high dielectric loss. Another way to improve or change the properties of BFN is to create solid solutions with it. For example, in [51], in solid solution BT-BFN obtained by a solid-state reaction route, a high dielectric constant, low dielectric loss, and a saturated ferroelectric hysteresis loop were obtained.

In the existing thematic literature, there are limited reports on the effect of La admixtures on the dielectric properties of BFN materials. The novelty of the present work is in analyzing the influence of a lanthanum admixture on the physical properties of the Ba_1−3/2*x*_La*_x_*(Fe_0.5_Nb_0.5_)O_3_ ceramics prepared by the solid-state sintering method at 1350 °C. The experiment aims to maintain high permittivity values and reduce dielectric loss and high electrical conductivity of the BFN*x*La material. The structural, morphological, and electrical measurements and DC electrical conductivity of the BFN*x*La samples were examined.

## 2. Materials and Methods

### 2.1. Technological Process

The lead-free compositions Ba_1−3/2*x*_La*_x_*(Fe_0.5_Nb_0.5_)O_3_ (BFN*x*La) with lanthanum admixtures (*x* = 0.00 to 0.06) were synthesized via the solid-state reaction method. Simple oxides, Fe_2_O_3_ (99.98% purity, Sigma-Aldrich, St. Louis, MO, USA), Nb_2_O_5_ (99.9% purity, Sigma-Aldrich, St. Louis, MO, USA), La_2_O_3_ (99.98% purity, Sigma-Aldrich, Steinheim, Germany), and barium carbonate BaCO_3_ (99.99% purity, POCH, Gliwice, Poland), were used in the technological process for obtaining the BFN*x*La ceramic materials. The input powders were weighed in stoichiometric proportions and mixed in a Fritsch planetary mill (Pulverisette-6, Idar-Oberstein, Germany) for 8 h. The wet milling was done in ethyl alcohol using zirconia-milling balls (the ball/powder weight ratio was 2/1) with a 250 rpm rotating speed of the planetary mill. Next, the powders were synthesized under the following condition: 1250 °C/4 h via the solid-state reaction method. The powder was pressed into compacts using a hydraulic press at a pressure of 300 MPa. Then, the compacts were ground to a powder and remixed. Then, the powder was pressed into compacts with a diameter of 10 mm and a thickness of 2 mm on a hydraulic press under a pressure of 300 MPa. The ceramic compacts were placed in a ceramic crucible in an Al_2_O_3_ environment and sintered using a pressure-less sintering method under a condition of 1350 °C/2 h (at a linear heating rate of 300 °C/h). After sintering, the BFN-type samples were polished (up to 1 mm thick) and then annealed at 750 °C for 15 min (to remove internal stress acquired during mechanical treatment). All BFN*x*La samples were obtained under the same conditions to compare the influence of the lanthanum admixture on the microstructure and electrophysical properties of the BFN-type material. Silver electrodes were applied to both surfaces of the ceramic sample for electrophysical tests using the firing method (850 °C/15 min).

### 2.2. Characterization

The XRD patterns of the BFN*x*La materials were derived using a Philips diffractometer (Panalytical, Eindhoven,, The Netherlands) with a CuK_α_ source and a graphite monochromator. All X-ray diffraction patterns were registered at RT in the 2*θ* range from 5° to 60°. Phase identification was performed based on data from the ICDD PDF-4 database (International Center for Diffraction Data Powder Diffraction Files). SEM microstructure of fracture ceramic samples, EDS (Energy Dispersive Spectrometry), and EPMA (Electron Probe Microanalysis) studies were carried out by a JEOL JSM-7100 TTL LV Field Emission Scanning Electron Microscope (JEOL Ltd., Tokyo, Japan). The SEM/EDS/EPMA tests were performed at a low vacuum and an accelerating voltage of 15 kV. For microstructure analysis, cross-sectional surfaces of ceramic samples were coated with gold using a Smart Coater DII-29030SCTR (JEOL Ltd., Tokyo, Japan). Elemental analysis of surfaces in SEM was performed using the EDS technique, which measures the energy and intensity distribution of X-ray signals generated by the electron beam striking the surface of the ceramic samples. The average grain size $\bar{r}$ was determined based on microstructural SEM images using ImageJ software (ImageJ 1.37v, LOCI, University of Wisconsin-Madison, WI, USA). Temperature-dependent measurements of dielectric properties for BFN*x*La ceramic samples were carried out using a QuadTech 1920 Precision LCR Meter (QuadTech, Maynard, MA, USA) in the temperature range from RT to 450 °C and in the *f* frequency range from 1 kHz to 1 MHz (in the heating cycle, heating rate of 3.0 deg/min). Uncertainty in the measurement of dielectric parameters, i.e., *C*_p_ capacitance and tan*δ* dielectric loss tangent, was ±0.25% and ±0.0025, respectively. Direct current electrical conductivity measurements were performed using a digit multimeter Keysight 34465A (Keysight Technologies, Inc., Santa Rosa, CA, USA) in the temperature range from RT to 450 °C. The measurement accuracy of the current was ±(0.1% + 100 pA).

## 3. Results and Discussion

### 3.1. Crystal Structure

**Figure 1:**
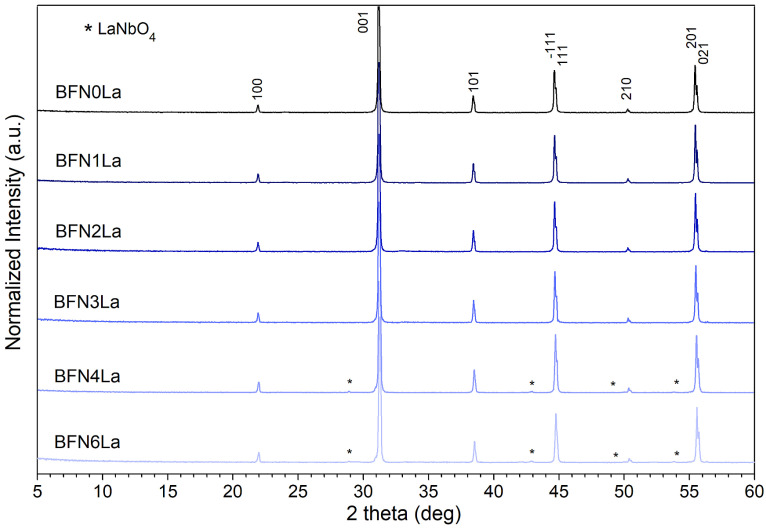
XRD patterns of the BFN*x*La ceramic samples at RT.

X-ray diffractograms of the BFN*x*La ceramic samples at RT are plotted in Figure 1. The X-ray tests revealed several clear peaks assigned to the BaFe_0.5_Nb_0.5_O_3_ perovskite phase, with card 04-008-1884 belonging to the cubic system (space group P*m*$\bar{3}$*m*). The sharpened peaks in the X-ray results are due to the BFN compound’s strong crystallinity. In the case of the BFN4La and BFN6La samples, the X-ray analyses also revealed the formation of a tiny amount of LaNbO_4_ secondary phase (pyrochlore lanthanum orthoniobate LaNbO_4_ with a monoclinic structure). In these compositions, it is related to a large amount of lanthanum admixture introduced into the base composition, the excess of which and high sintering temperature favor the combination of lanthanum with niobium, forming an additional admixture phase. The electronegativity of niobium (1.6) is much greater than that of lanthanum (1.1). In different La–Nb–O compounds, La^3+^ can decrease the orbital overlap by easing some charge densities of Nb–O bonds, leading to easily enabling a redox reaction of Nb^5+^/Nb^4+^ [52]. A foreign phase in La-doped BFN was also revealed in the [53,54] due to an excessive substitution of La^3+^. It was also noticed that the volume of the unit cell decreases with an increasing lanthanum concentration due to the large disproportion of ionic radii of La^3+^ (1.36 Å) and Ba^2+^ (1.61 Å).

The crystal lattice distortion of the BFN*x*La ceramic samples can be predicted based on the tolerance factor formula [55] of ABO_3_-type perovskite structures expressed as Equation (1):

(1) $$\gamma =\frac{r_{A}+r_{O}}{\sqrt{2}\left(r_{B}+r_{O}\right)\;},$$

where *r_A_*, *r_B_*, and *r_O_* denote the ionic radius of atoms A, B, and O, respectively (in Å). The calculated tolerance factor based on the standard ionic radii of the tested samples is tabulated in Table 1 along with the corresponding ionic radii used. The tolerance factor decreases slightly with an increase in lanthanum in the composition of BFN and suggests a lattice rearrangement of atoms. That is, it can be stated that the lattice is stabilized in terms of symmetry and atomic arrangement because of the observed decrease in effective strain [55].

### 3.2. Microstructure

**Figure 2:**
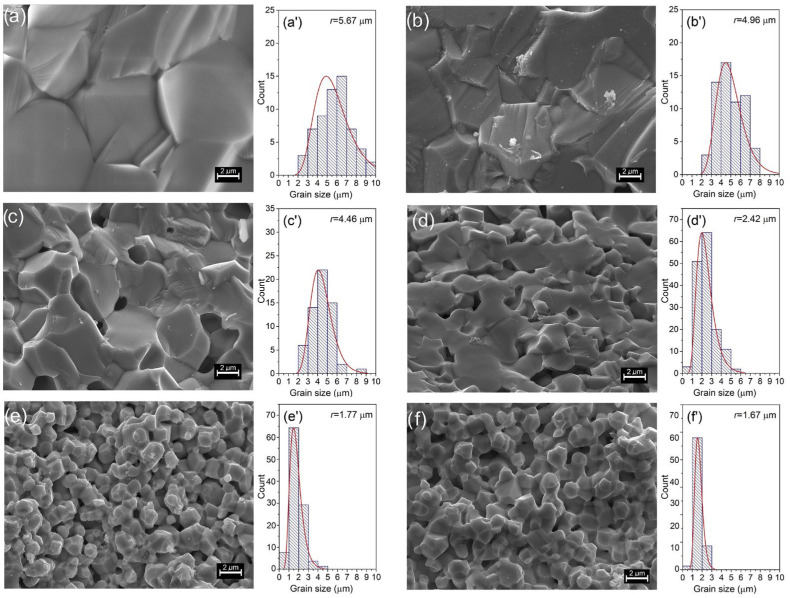
SEM images of the microstructure of fractures of the ceramic samples: (**a**,**a′**) BFN0La, (**b**,**b′**) BFN1La, (**c**,**c′**) BFN2La, (**d**,**d′**) BFN3La, (**e**,**e′**) BFN4La, and (**f**,**f′**) BFN6La, respectively. Next to it, grain size distribution diagrams.

The microstructural SEM images of the BFN*x*La sample series show a change in surface morphology with increased lanthanum in the elemental BFN composition (Figure 2). The BFN ceramics microstructure is characterized by a compact structure with close-packed big grains (the average grain size $\bar{r}$ is 5.67 μm) and visible grain boundaries. The sample is broken down in two ways, i.e., along the inter-grain boundaries and inside the grain. It is proven that the mechanical strength of the ceramic grains is equally high both at the grain boundary and inside the grain.

The introduction of lanthanum into the BFN material reduces the average grain size in the microstructure, changing the microstructure from coarse-grained to fine-grained. For BFN1La’s composition, the microstructure is similar to an undoped BFN material with the same strength properties but a smaller average grain size ($\bar{r}$ = 4.96 μm). As the lanthanum content in BFN*x*La increases, the microstructure becomes more and more fine-grained, and the glued grains form more giant conglomerates. A blurring of the boundaries between grains also starts to occur. For the highest lanthanum content in the BFN materials (BFN4La and BFN6La), the microstructure is characterized by the finest grain, with strong sintering and unsharp grain boundaries. The average grain size $\bar{r}$ is 1.77 μm (for BFN4La), whereas for BFN6La it is 1.67 μm. Similar results on the influence of a lanthanum admixture on the microstructure of BFN materials were obtained in [53]. The average grain size decreased as the La content increased in the BFN because the trivalent-ion La^3+^ occupied A sites (Ba), favoring the formation of donor imperfections Ba_La_ [53]. However, lanthanum ions can also fill the B sites (Fe/Nb) and act as acceptors. Low doping levels lead to compensation for electric and barium vacancies in the BFN [53]. The appearance of the microstructure for these compositions is also determined by forming a secondary phase, which was also confirmed by X-ray studies for BFN4La and BFN6La samples.

### 3.3. EDS and EPMA Tests

**Figure 3:**
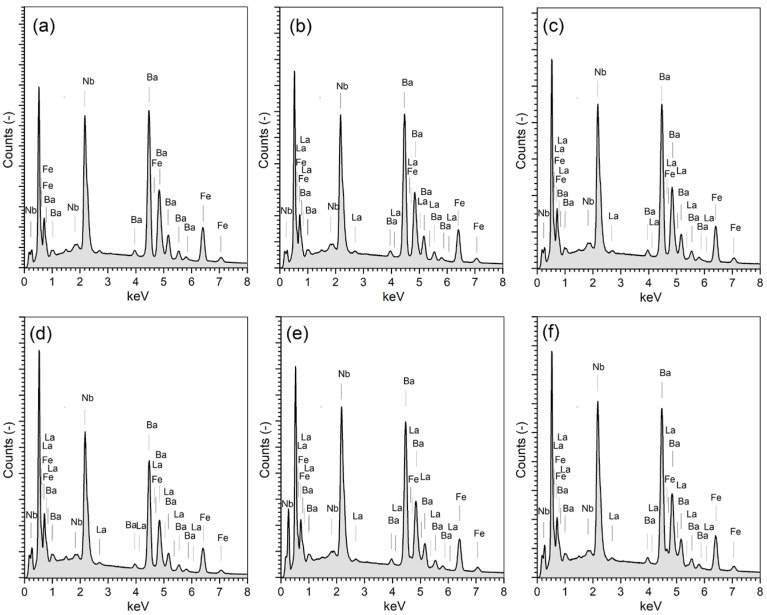
The EDS analysis of chemical elements of the ceramic samples: (**a**) BFN0La, (**b**) BFN1La, (**c**) BFN2La, (**d**) BFN3La, (**e**) BFN4La, and (**f**) BFN6La, respectively.

The energy dispersive spectroscopy (EDS test) of the BFN*x*La materials is depicted in Figure 3 and was performed on micro-areas on the cross-section surface of the ceramic samples (the results are the average value of five randomly selected sample areas). The EDS test allows for a qualitative analysis of the distribution of elements based on automatic scanning of a specific micro-area of the surface (point analysis) and a qualitative study consisting of identifying individual elements in the characteristic X-ray spectrum. The results of the EDS analysis confirmed both the qualitative and quantitative content of the individual components of the BFN*x*La ceramic samples without the presence of foreign elements (inclusions). In the case of the undoped BFN ceramics, the EDS study showed a slight reduction in barium and iron, with a slight increase in niobium. The EDS tests for the BFN*x*La ceramic samples revealed a slight decrease in barium and iron, with an increase in niobium and lanthanum. However, their deviation from the assumed composition is within the permissible error (Table 2). The EDS tests have also shown lower oxygen content in all samples, resulting from its loss during the high-temperature technological process.

**Figure 4:**
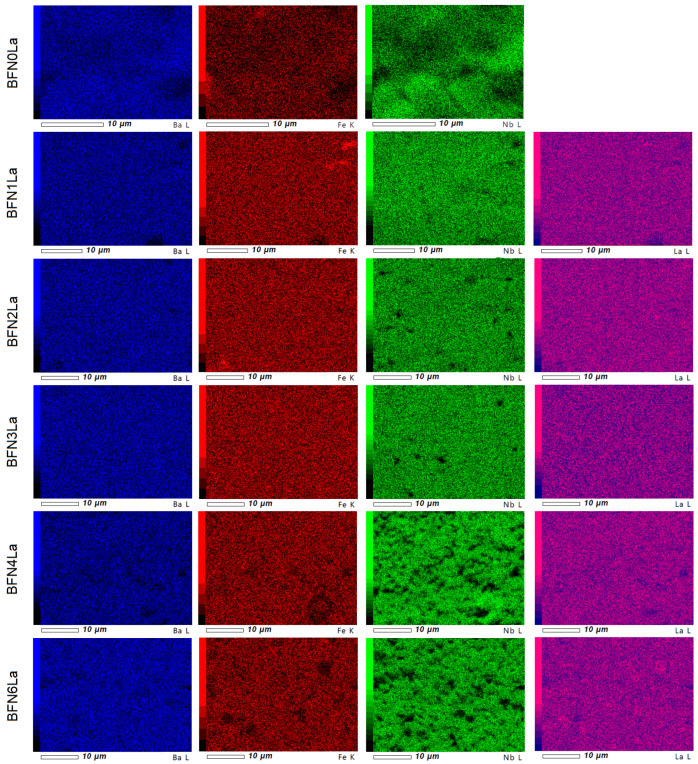
EPMA test results for the BFN*x*La ceramics.

The electron probe microanalysis (EPMA maps) of the individual element distribution of the BFN*x*La ceramic samples are depicted in Figure 4. The analysis showed a homogeneous distribution of the elements barium, iron, and lanthanum in the sample volume (made on the cross-section of the sample). In the case of more significant depressions of the sample surface, reading the probe was difficult—dark areas were visible on the EPMA maps. These anomalies occurred especially in the detection of niobium.

### 3.4. DC Electric Conductivity

**Figure 5:**
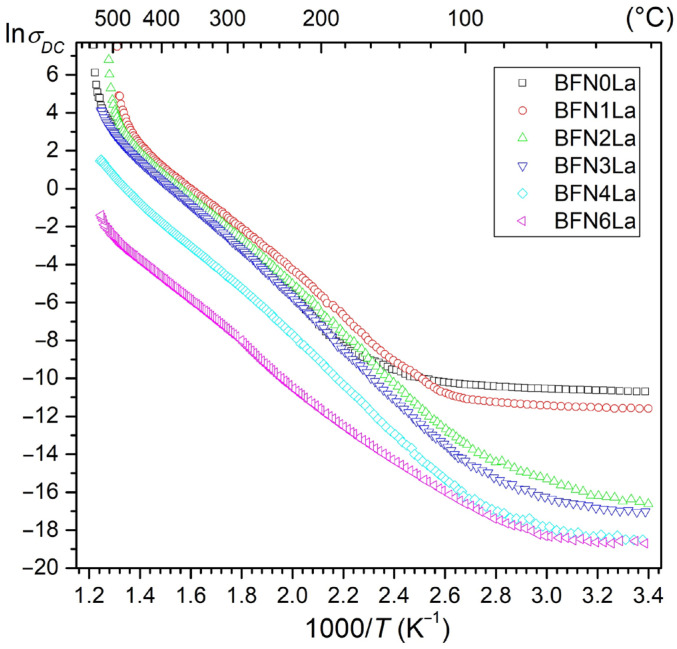
The lnσ_DC_(1000/*T*) relationship of the BFN*x*La ceramic samples.

The BFN*x*La materials at RT have an average value of the DC electrical conductivity, i.e., 1.82 × 10^6^ Ωm (for BFN0La), 2.14 × 10^6^ Ωm (for BFN1La), 4.60 × 10^6^ Ωm (for BFN2La), 5.92 × 10^6^ Ωm (for BFN3La), 6.64 × 10^6^ Ωm (for BFN4La), and 6.85 × 10^6^ Ωm (for BFN6La). Figure 5 shows the dependencies of ln*σ*_DC_(1000/*T*) for the BFN*x*La ceramic samples. The graph shows areas with different slopes of the ln*σ*_DC_(1000/*T*) curves, i.e., at lower and higher temperatures (occurring in various temperature ranges, depending on the amount of lanthanum in the composition). At lower temperatures, with increasing temperature, the growth in electrical conductivity is negligible. Above 150 °C, the conductivity rises faster but linearly, and after exceeding approximately 450 °C, the increase in conductivity is more pronounced.

Iron is characterized by oxidizing properties that occur at high temperatures. In the case of undoped BFN, at high temperatures, the iron cation Fe^3+^ is quite easily reduced to Fe^2+8^ according to the pattern.

Fe^3+^ + *e*^−^ → Fe^2+^. (2)

In the pure and stoichiometric state, in which the Fe and Nb ions are in the 3+ and 5+ valence states, appropriately, BFN materials should behave with insulator properties. However, the technological process is carried out at very high temperatures, which favors the formation of oxygen vacancies and charge carriers (electrons and holes). Emerging defects of the unit cell network contribute to a complex system of conduction mechanisms in BFN ceramic materials. The following reaction can present the occurrence of oxygen vacancies at high temperatures:

(3) $$O_{O}\to \frac{1}{2}O_{2}+V_{O}^{\prime \prime }+{2e}^{-}.$$

In such a case, the two liberated electrons can be captured by the Fe^3+^ and Nb^5+^ ions [56,57]. Charge transport by electron hopping takes place using an oxidation-reduction numerous process between the Fe and Nb ions, which can be written in the form of the chain notation Fe^2+^–O–Fe^3+^, Nb^4+^–O–Nb^5+^, Fe^2+^–O–Nb^5+^, Nb^4+^–O–Fe^3+^, or their combinations [56,57]. Oxygen vacancies and cations with lowered valency are donor centers (Fe^2+^ and Nb^4+^) and lead to the hopping mechanism of conductivity, creating extrinsic n-type conduction in undoped BFN ceramics. On the other hand, oxygen vacancies make generating holes easier and lead to p-type conductivity [56]. The lowest values of DC electric conductivity in the ferroelectric and paraelectric phases are for the composition with the most significant amount of lanthanum admixture (BFN6La). In contrast, the highest ones occur for undoped BFN (BFN0La). Based on the slope of the curves ln*σ*_DC_(1000/*T*) and Arrhenius’ Equation (4), the activation energies *E_a_* were calculated (in these two temperature regions with different slopes of the waveforms).

(4) $$\sigma =\sigma \exp\left(\frac{E_{a}}{k_{B}T}\right),$$

where *σ*_0_*—*pre-exponential factor, *k_B_—*Boltzmann’s constant, *T—*absolute temperature, and *E_a_—*the activation energy. The calculated activation energy *E_a_* values (Table 1) are higher than energy values connected with a change in iron valence Fe^3+^⇔Fe^2+^ (0.1 eV). For BFN*x*La materials, in higher temperatures, the activation energy values (*E_a_*) are higher in comparison with the lower-temperature region, which is characteristic of materials with a perovskite structure [58]. La admixing of the BFN materials in the ferroelectric phase increases *E_a_* values and decreases them in the paraelectric phase. The most significant changes in *E_a_* are caused by admixture with La^3+^ (Table 1). The activation energy in BFN-type materials is mainly connected with the charge carriers’ mobility. This indicates a hopping mechanism of the conductivity associated with the occurrence of oxygen vacancies and valency changes of Fe and Nb [57].

### 3.5. Dielectric Measurement

**Figure 6:**
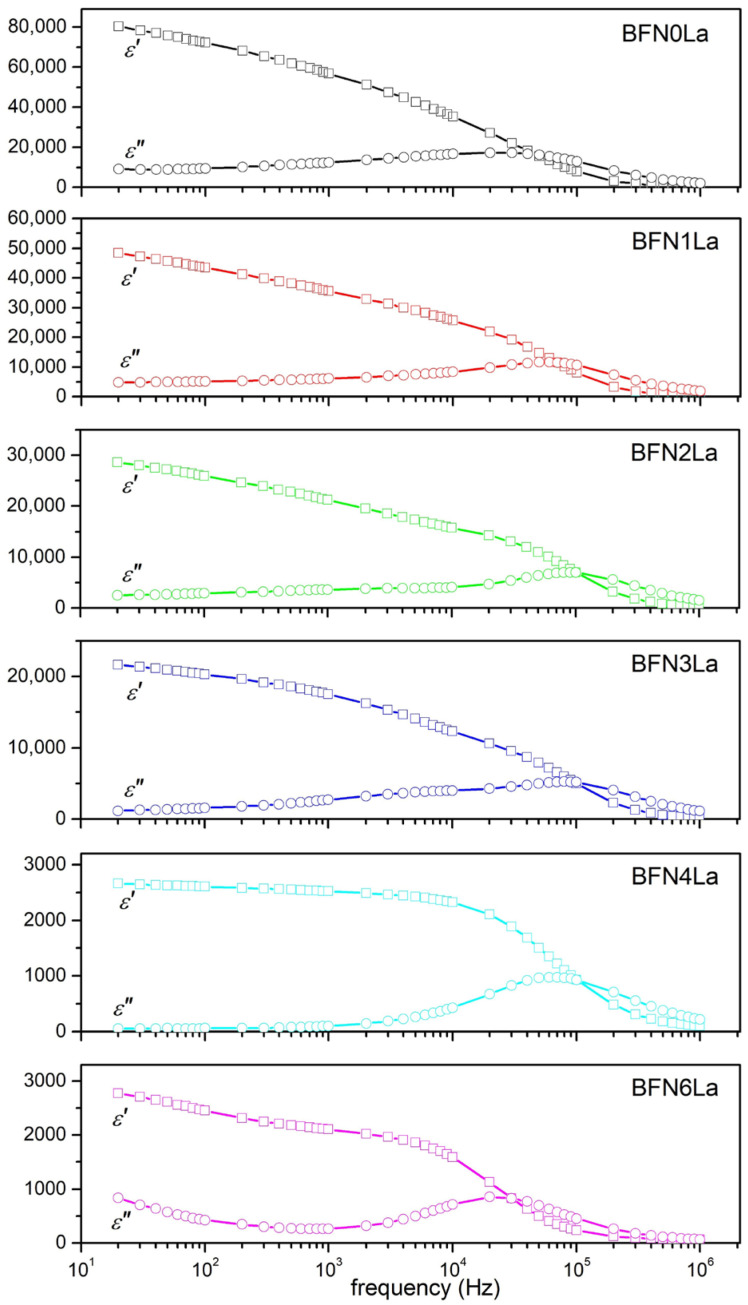
Frequency dependence of real *ε*′ and imaginary *ε*″ parts of the dielectric constant of the BFN*x*La ceramics.

Figure 6 shows the frequency dependence of the real *ε*′ and imaginary *ε*″ parts of the dielectric constant (and in the form of connected charts in the Supplementary Data) for a series of the BFN*x*La ceramic samples measured at room temperature. All BFN*x*La samples show a Debye-like relaxation behavior [59], and at lower frequencies, the occurring frequency dispersion of the dielectric constant becomes weaker with increasing admixtures of lanthanum. The *ε*′ values gradually decrease with increasing frequency, and when the maximum *ε*″ occurs, *ε*′ values decrease more rapidly. At higher frequencies (above 400 kHz), the *ε*′ value stabilizes. When the La admixture increases in the BFN*x*La, the relaxation peak shifts to a lower frequency (Figure S1, Supplementary Materials). Also, the dielectric constant of the BFN*x*La decreases significantly with the increase in lanthanum content. For example, at 1 kHz, *ε*′ is 56,750 for BFN0La, 35,550 for BFN1La, 21,226 for BFN2La, 17,550 for BFN3La, 2523 for BFN4La, and 2104 for BFN6La.

The dielectric constants for all the samples show dispersion at lower frequencies, and they will stabilize at higher frequencies. This is related to the Maxwell–Wagner type interfacial polarization model [60] based on Koop’s theory [61,62]. In practice, the polycrystalline ceramic sample exhibits a heterogeneous microstructure comprising semiconducting grains separated by insulating grain boundaries. The inhomogeneity of the ceramic sample arising during sintering at high temperatures includes, among others, porosity, grain size inhomogeneity, impurities, interfacial defects, vacancies, etc. Oxygen vacancies in the ceramic sample create additional space charges that accumulate on the boundary between the sample and the electrode [63]. Consequently, the total polarization of the sample will contain a component originating from the near-electrode layer. At low frequencies, the created space charges can move longer distances in the sample, increasing the electronic polarization. This results in high dielectric constant values. Increasing the frequency of the applied field reduces the time for charge carriers to move in the direction of the field; therefore, the polarizability decreases, and consequently, the dielectric constant decreases [63]. In ceramic materials, there is a correlation between microstructure and dielectric properties, i.e., the dielectric constant is linearly proportional to the average value of the grain size and inversely proportional to the grain boundary thickness [63,64]. In the BFN*x*La, the dielectric constant decreases as the grain size in the microstructure decreases due to lanthanum doping. At higher amounts of La, the grain boundary thickness increases due to the presence of the foreign phase, the presence of which further reduces the dielectric constant. The authors of [53] also confirmed the negative impact of forming a foreign phase of the BFN-type materials on the dielectric constant.

Thus, in the BFN material, one of the reasons for the decrease in the dielectric constant and dielectric loss values with increasing La admixture is the reduction of the grain size in the microstructure. This is consistent with the research results presented in [65] and explained by the conduction mechanism. In the microstructure of ceramic materials, grain boundaries show high resistance. This means more energy is required to move charge carriers, increasing energy losses. According to Debye’s relaxation theory, ceramic materials containing iron exhibit a maximum of *ε*″ dielectric loss when the frequency of electron jumping between Fe^2+^ and Fe^3+^ ions is consistent with the frequency of the applied field, which is attributed to domain wall resonance [66]. For all BFN*x*La compositions, in the *ε*″(*f*) plots, Debye-like relaxation peaks appear, followed by a decrease in the *ε*″ value with a further increase in frequency (Figure 6). At higher frequencies, *ε*″ decreases due to slowing down the movement of domain walls. Similar research results were obtained by the authors of [59] for some other compounds, e.g., ACu_3_Ti_4_O_12_ (A = Ca, Bi_2/3_, Y_2/3_, La_2/3_) in the ranges of 10^−1^–10^6^ Hz, but obtaining much lower *ε*′ and *ε*″ values.

**Figure 7:**
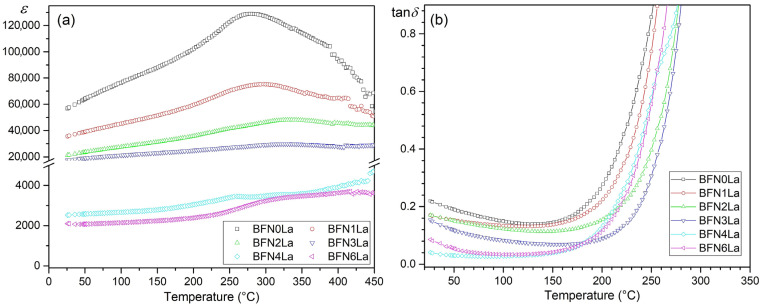
Temperature dependencies of the (**a**) permittivity and (**b**) dielectric loss factor for BFN*x*La ceramics measured at 1 kHz.

Figure 7a presents the temperature dependencies of permittivity for the series of BFN*x*La samples (for 1 kHz). BFN*x*La materials show high permittivity values at RT and the *T_m_* temperature, and the phase transition occurs at a wide range of temperatures. Previous studies [43,67] have shown that BFN does not have an acute phase transition characteristic for PZT-type materials, but that the phase transition from ferroelectric to paraelectric phase occurs over a wide temperature range. In our experiment, dielectric studies of BFN*x*La also confirmed the diffuse phase transition, but much higher permittivity values were obtained. A wide phase transition temperature range is associated with disorder cation distribution (in the B-site) occurring in perovskite-type materials. In the case of BFN materials, Fe^3+^ and Nb^5+^ ions are randomly located in B places of the crystal structure. Due to the presence of larger Fe^3+^ cations (0.78 Å) and smaller Nb^5+^ (0.74 Å) cations, a more extensive “rattling space”, it occurs in a unit cell [67]. In the case of BFN*x*La materials, the ionic radius of lanthanum also allows the substitution of excess La in the B position of the compound. Appearance in BFN composition fluctuation, i.e., a disturbance in the distribution of ions at the B site in the perovskite unit cell, causes the formation of micro areas with different local Curie temperatures and consequently broadens the temperature range of phase transition of ceramics. Temperature tests of dielectric properties for the analyzed series of BFN*x*La samples for the frequency range 1 kHz–1 MHz are summarized in the Supplementary Data (Figure S2, Supplementary Materials). In the case of the BFN*x*La for *x* from 0.00 to 0.03, a decrease in the permittivity is observed with increasing frequency. A clear shift in maximum permittivity towards higher temperatures indicates typical relaxation behavior [54]. In BFN*x*La compositions above *x* = 0.04, a foreign phase disturbs this phenomenon. In [54], temperature dielectric tests of La-doped BFN were presented, which showed a clear phase transition. However, comparing the results for individual compositions from [54] with our results, it can be concluded that our samples have much higher permittivity values. In [53], in dielectric tests, high permittivity values are not accompanied by a clear phase transition of BFN-type materials.

Thus, the admixture of lanthanum introduced into the base BFN composition reduces the permittivity values in the entire measurement area. It is especially visible in the case of BFN4La and BFN6La compositions, where a secondary phase is formed apart from the fine-grained microstructure. There is a similar trend in the effect of lanthanum doping in BFN on the dielectric loss factor. Figure 7b presents tan*δ*(*T*) plots for all BFN*x*La ceramic samples in the temperature range from RT to 450 °C for 1 kHz. At RT, the tan*δ* values of the BFN*x*La ceramic samples are relatively low, i.e., 0.22 (for BFN0La), 0.18 (for BFN1La), 0.17 (for BFN2La), 0.15 (for BFN3La), 0.04 (for BFN4La) and 0.08 (for BFN6La). It can be observed that initially the tan*δ* values decrease with increasing temperature, and growth is rapid above 150 °C. The mobility of charge carriers increases with temperature, which increases the polarization and leads to high dielectric loss. The observed higher value of dielectric loss at high temperatures is due to charge accumulation at grain boundaries [68]. As the amount of La in the BFN*x*La increases, the dielectric loss factor decreases (the lowest tan*δ* values have the compositions of BFN4La and BFN6La).

It is common knowledge that BFN materials have high dielectric loss factor values (tan*δ*) [43,46]. An increase in temperature above 200 °C causes a large increase in the tan*δ* value due to a significant increase in the electrical conductivity of the BFN material. Such a tremendous increase is attributed to this space charge polarization conduction thermally, usually observed in ferroelectric materials (especially in iron-containing ceramic materials). The appearance of space charge polarization is mainly caused by the partial reduction of Fe^3+^ to Fe^2+^, and using the Kröger–Vink notation, the defect reaction can be written as the following relationship [69]:

(5) $${Fe}_{Fe}^{x}\to {Fe}_{Fe}^{\prime }+h^{•}.$$

The following conduction mechanisms may be related to the ferroelectric properties of the ceramic material [56]. In the first mechanism (occurring below *T*_m_), lattice strains associated with order–disorder changes originate during the increase of the temperature, inducing conduction by small polarons (electron and/or hole-phonon interactions) where the carrier transport takes place via hopping charge. The second mechanism is ionic conductivity, a characteristic conduction mechanism in ferroelectric materials at high temperatures [56].

## 4. Conclusions

Lead-free Ba_1−3/2*x*_La*_x_*(Fe_0.5_Nb_0.5_)O_3_ (when *x* = 0.0 to 0.6) ceramic prepared through a solid-state reaction method was found to have a perovskite-type cubic structure with the space group P*m*$\bar{3}$*m*. The effect of La content on the microstructure and dielectric properties of BFN ceramics was investigated. Starting from the compositions BFN*x*La for *x* = 0.04, a small pyrochlore LaNbO_4_ phase was created during the technological process. The average grain size in the microstructure decreased with the increase in La content in the BFN*x*La ceramic samples. The BFN*x*La compositions in which the presence of the second phase was revealed had the smallest grains.

In undoped BFN, the oxidation phenomenon occurring at high temperatures results in the excessive formation of oxygen vacancies. Introducing an admixture of lanthanum causes their adequate compensation by forming cationic vacancies, consequently reducing the electrical conductivity of the BFN*x*La ceramic samples. In BFN*x*La ceramic samples, there was a decrease in dielectric constant and dielectric loss values as a function of La doping. The shifting of phase transition temperature towards a higher temperature with the frequency hike confirmed the compounds’ relaxor behavior. BFN*x*La samples for *x* from 0.01 to 0.03 showed equally high permittivity values (compared to undoped BFN) but gained lower dielectric loss and electric conductivity. The high permittivity of the BFN*x*La material predisposes it for energy storage applications, while lower tan*δ* values, especially in the higher frequency area, are beneficial in constructing magnetically tunable filters, resonators, and oscillators.

## Figures and Tables

**Table 1:** Electrophysical properties of the BFN*x*La materials.

Parameter	BFN0La	BFN1La	BFN2La	BFN3La	BFN4La	BFN6La
*γ*	0.8367	0.8364	0.8360	0.8356	0.8355	0.8326
*ρ* (Ωm)	1.82 × 10^6^	2.14 × 10^6^	4.60 × 10^6^	5.92 × 10^6^	6.64 × 10^6^	6.85 × 10^6^
$\bar{r}$ (μm)	5.67	4.96	4.48	2.42	1.77	1.67
*ε′* at RT	56,750	35,550	21,226	17,550	2523	2104
*ε″* at RT	12,485	6400	3609	2632	101	168
tan*δ* at RT	0.22	0.18	0.17	0.15	0.04	0.08
*E_a_* at I (eV)	0.048	0.064	0.302	0.153	0.160	0.096
*E_a_* at II (eV)	1.075	0.968	1.042	1.108	1.059	0.898

**Table 2:** Theoretical and experimental percentage of the individual components of the BFN*x*La.

Element	BFN0La		BFN1La		BFN2La		BFN3La		BFN4La		BFN6La	
	TH	EX	TH	EX	TH	EX	TH	EX	TH	EX	TH	EX
Ba	52.88	52.61	52.35	52.25	51.82	51.24	51.28	51.02	50.75	51.11	49.69	49.46
Fe	10.75	10.40	10.75	10.57	10.75	10.53	10.75	10.64	10.75	10.32	10.75	10.54
Nb	17.88	18.75	17.89	18.25	17.88	18.56	17.88	18.13	17.88	17.95	17.88	18.01
La	-	-	0.53	0.62	1.07	1.29	1.61	1.8	2.14	2.23	3.21	3.66
O	18.48	18.24	18.48	18.31	18.48	18.38	18.48	18.41	18.48	18.39	18.47	18.33

TH—theoretical (mass %), EX—experimental data (mass %).

## Data Availability

The original contributions presented in the study are included in the article/Supplementary Materials, further inquiries can be directed to the corresponding authors.

## Supplementary Materials

The following supporting information can be downloaded at: https://www.mdpi.com/article/10.3390/ma17153666/s1: Figure S1, Cumulative chart of the frequency dependence of real *ε*′ and imaginal *ε*″ part dielectric constant for BFN*x*La ceramics; Figure S2, Temperature dependence of permittivity and dielectric loss factor tan*δ* for BFN*x*La ceramics.
