# Supplementary material for: The Influence of Lanthanum Admixture on Microstructure and Electrophysical Properties of Lead-Free Barium Iron Niobate Ceramics
Source: Materials (Basel). 2024 Jul 25;17(15):3666. doi: 10.3390/ma17153666 (PMC11312510; doi:10.3390/ma17153666)
Supplement: Supplementary file 1 [file materials-17-03666-s001.zip › materials-3112839-supplementary.pdf]

Supplementary Materials

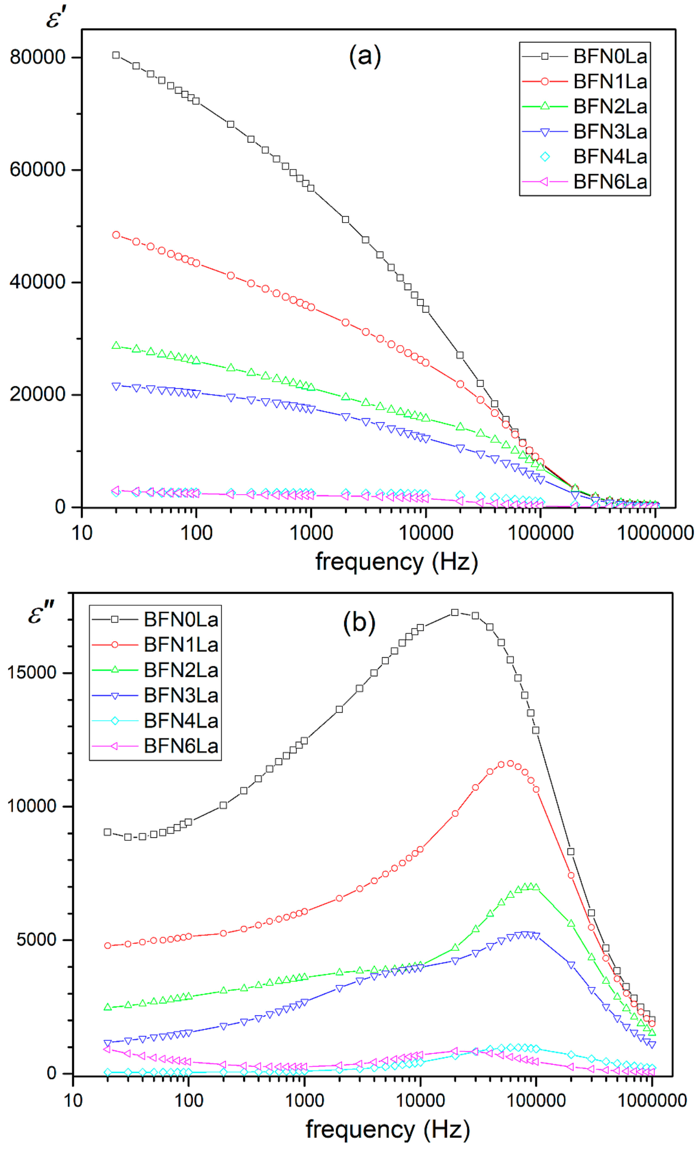

Figure S1 Cumulative chart of the frequency dependence of (a) real ε' and (b) imaginal ε'' part dielectric constant for BFNxLa ceramics.

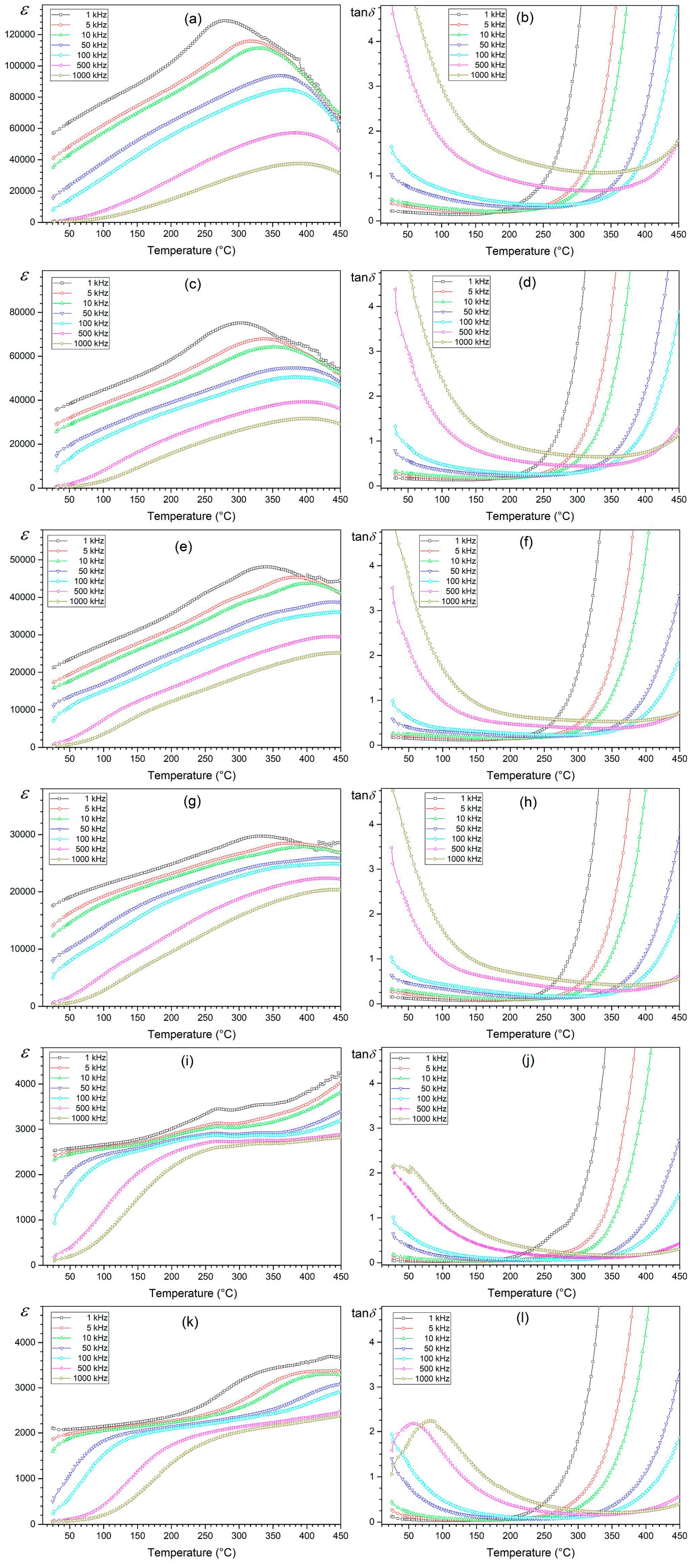

Figure S2 Temperature dependence of (a, c, e, g, i, k) permittivity and (b, d, f, h, j, l) dielectric loss factor tanδ for BFNxLa ceramics; (a–b) BFN0La, (c–d) BFN1La, (e–f) BFN2La, (g–h) BFN3La, (i–j) BFN4La and BFN6La (k–l); tests for 1, 5, 10, 50, 100, 500, 1000 kHz.
